# Dr. Charles Coates

**Published:** 1909-04-03

**Authors:** 


THE death is announced of Dr. Charles Coates, who died on March 23 at his residence at Bath, at the age of 83. Dr. Coates, who had retired from practice about ten years, was the oldest member of the medical profession in Bath. He was for many years, and up to the time of his death, a governor of the Royal Mineral Water Hospital. He was connected by marriage to the family of the sixth Earl of Buckingham. Dr. Coates liberally supported the Bath charitable institutions. He was honorary consulting physician to the Royal School for Daughters of Officers of the Army, Lansdown, a trustee of Partis College, and also of Holburne Museum. He gave £1,000 to the Blue Coat School and a similar sum to the Royal College of Physicians to found a prize. He became a Fellow of the Royal College of Physicians, Edinburgh, in 1857, and of the Royal College of Physicians, London, in 1873. He was formerly resident clinical assistant at the Brompton Hospital for Consumption.

